# Supplementary material for: Collaborative networks enable the rapid establishment of serological assays for SARS-CoV-2 during nationwide lockdown in New Zealand
Source: PeerJ. 2020 Sep 3;8:e9863. doi: 10.7717/peerj.9863 (PMC7474877; doi:10.7717/peerj.9863)

Supplementary Figure 1. Serum IgG reactivity of pre-pandemic healthy adult controls with human coronavirus S proteins. ELISA were performed with sera (n=31) against S1 proteins of one β- (HKU1) and two α-HCoVs (NL63 and 229E) as well as S1 and Spike proteins of SARS-CoV2. The cutoff of OD >0.2 is indicated for reference to SARS-CoV2 spike protein ELISA. IVIG is indicated as pink squares.

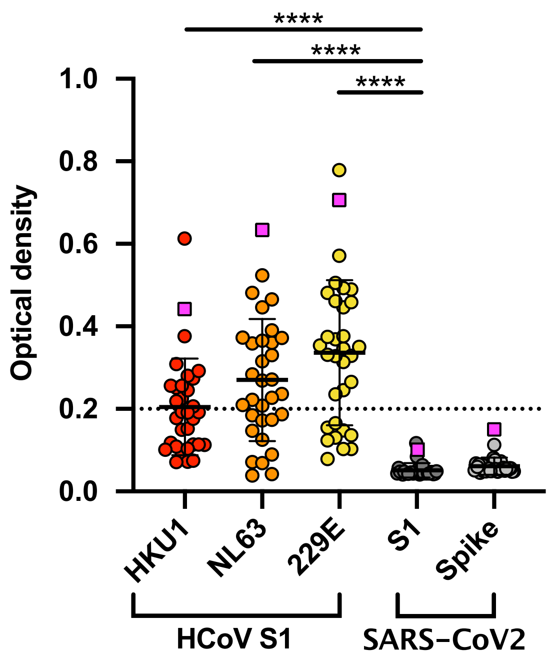

Supplement: Supplemental Information 2 — ELISA were performed with sera (n = 31) against S1 proteins of one β- (HKU1) and two α-HCoVs (NL63 and 229E) as well as S1 and Spike proteins of SARS-CoV2. The cutoff of OD >0.2 is indicated for reference to SARS-CoV2 spike protein ELISA. IVIG is indicated as pink squares. [file peerj-08-9863-s002.pdf]
